# Electron Microscopy for Rapid Diagnosis of Emerging Infectious Agents^1^

**DOI:** 10.3201/eid0903.020327

**Published:** 2003-03

**Authors:** Paul R. Hazelton, Hans R. Gelderblom

**Affiliations:** * University of Manitoba, Winnipeg, MB, Canada; † Robert Koch-Institut, Berlin, Germany

**Keywords:** Rapid diagnostic electron microscopy, morphological diagnosis, negative staining, particle enrichment, bioterrorism, agri-terrorism, quality control, synopsis

## Abstract

Diagnostic electron microscopy has two advantages over enzyme-linked immunosorbent assay and nucleic acid amplification tests. After a simple and fast negative stain preparation, the undirected, “open view” of electron microscopy allows rapid morphologic identification and differential diagnosis of different agents contained in the specimen. Details for efficient sample collection, preparation, and particle enrichment are given. Applications of diagnostic electron microscopy in clinically or epidemiologically critical situations as well as in bioterrorist events are discussed. Electron microscopy can be applied to many body samples and can also hasten routine cell culture diagnosis. To exploit the potential of diagnostic electron microscopy fully, it should be quality controlled, applied as a frontline method, and be coordinated and run in parallel with other diagnostic techniques.

In late September 2001, a letter containing spores of *Bacillus anthracis* arrived at a publishing house in Palm Beach, Florida, and resulted in the death of one employee from inhalation anthrax. Over the next 6 weeks, similar letters were delivered to television news centers in New York City and government offices in Washington, D.C. Ultimately >32,000 suspected exposures and five deaths were recorded in the United States. The collateral spread of exposure to spores was a sobering reminder of the bioterrorism attack scenario hypothesized by O’Toole (*1*).

Today, technology allows genetic engineering of potentially devastating agents such as modified ectromelia virus (*2*), the weaponizing variola virus (former USSR) (*3*), the long distance dispersal of yellow fever–infested mosquitos (United States) (*4*), and the weaponizing of anthrax spores by many nations. The ease with which the recent anthrax attacks were delivered indicates that unsophisticated methods are still effective. Thus, the most potent defenses remain rapid identification of the event and agent, treatment of the victims, and containment of infection. Successful outbreak management depends on early recognition of a suspected infectious case by the primary care physician and obtaining an accurate, timely laboratory diagnosis. An unexpected temporal or geographic cluster of illness of apparently infectious nature or an unusual age distribution of pneumonia with respiratory failure, intradermal hemorrhage, or chickenpox-like illnesses may indicate infection caused by a novel agent or a bioterrorist act. Similarly, the sudden appearance of vesicular lesions or respiratory illness in farm animals may be evidence of an emerging disease, a possible zoonosis, or an agriterrorist act. While recent studies suggest that health-care systems are ill prepared to treat victims and contain the spread of an infectious agent (*5*), the performance of physicians, epidemiologists, and diagnostic specialists in identifying outbreak-associated agents as diverse as Nipah virus (*6*) and gastroenteric agents (*7*) indicate that identification of an outbreak and its associated agent may be done rapidly and successfully.

Electron microscopic diagnosis is uniquely suited for rapid identification of infectious agents. A specimen can be ready for examination and an experienced virologist or technologist can identify, by electron microscopy, a viral pathogen morphologically within 10 minutes (*8*). We describe the role of transmission electron microscopy in viral diagnosis and outbreak management; methods for specimen collection, preparation, and examination; laboratory safety and quality control; and the differential morphologic diagnosis of infectious agents. In addition, an Appendix lists support facilities that provide electron microscopic diagnostic service.

## Role of Electron Microscopy in Virus Identification

The first electron micrograph of poxvirus was published in 1938. In 1941, immunologic procedures were first used in electron microscopic studies of tobacco mosaic virus (*9*), and electron microscopy was introduced successfully in the differential diagnosis of smallpox and chickenpox infections in the late 1940s (*10*,*11*). With the introduction of negative staining in the late 1950s (*12*) and the wider availability of electron microscopes, electron microscopy (as a catchall method) became essential in characterizing many new isolates detected in diagnostic cell cultures and clinical samples, e.g., stool, urine, and biopsied specimens (*7*,*13*–*16*). Pattern recognition, i.e., information on size and particle morphology, leads to rapid identification of infectious agents. The initial classification of many agents was therefore based on a combination of morphology and genome structure. Currently, >30,000 different viruses comprising 56 separate families have been identified, and humans have been found to host 21 of the 26 families specific for vertebrates (*17*). The distinct morphology of members of different viral families usually allows an agent to be assigned to a particular family. This morpho-diagnosis, combined with clinical information is, in most cases, sufficient to permit a provisional diagnosis or rule out a more serious infection and to initiate treatment and containment protocols without waiting for other test results.

**Figure 1:**
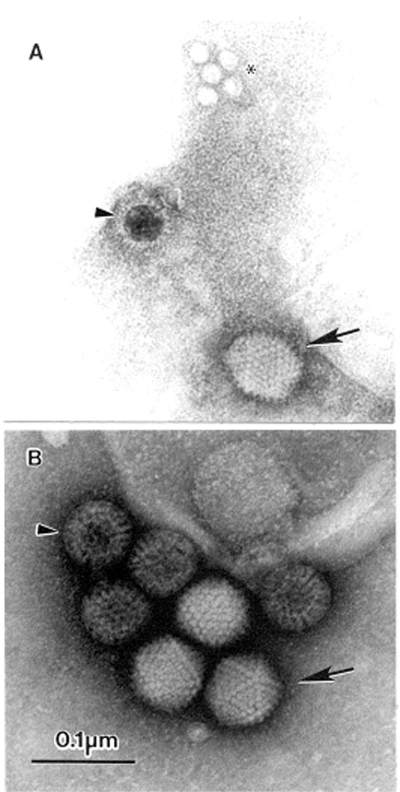
The open view of diagnostic electron microscopy. A. Multiple agents observed in a fecal sample from a pediatric patient with diarrhea. A 10% suspension was prepared in distilled water, cleared by low-speed centrifugation followed by 5 minutes at 15,000 x *g* in a bench top centrifuge, and centrifuged directly to the grid using an Airfuge EM-90 rotor (Beckman, Palo Alto, CA): adenovirus-(→), incomplete rotavirus-particle (>), and small round featureless particles, probably adeno-associated virus (ρ) phosphotungstic acid stained. B. Double infection with adenovirus (→) and complete rotavirus particles (>), in the stool of a 1-yearold child. The sample was suspended 1:3 in distilled water, cleared by low-speed centrifugation, and prepared for examination by the two-step method. Aqueous uranyl acetate stained. Bar = 100 nm.

Because electron microscopy is not suitable for screening large numbers of samples, many alternate immunologic and molecular methods have been developed on the basis of nucleic acid amplification techniques. While immunologic tests have almost unlimited throughput, the high specificity of these assays may result in failure to identify etiologic agents with different antigenic determinants. Further, reagents may not currently exist that would permit complete immunologic testing (*18*,*19*). Even when an immunologic test is appropriate for the etiologic agent, the sensitivity may only equal that of electron microscopy (*20*,*21*). Nucleic acid amplification techniques have similar limitations. They are more sensitive but are only capable of identifying the presence of genomic material for previously identified agents. Although primers exist that will permit amplification of most enteroviruses (*22*,*23*), few multiplex systems can identify all genotypes and serotypes within, or between, the different families of viruses that infect humans (*22*,*24*,*25*). Further, mutations in the primer target region may negate the effectiveness of primers. Because nucleic acid amplification techniques will not identify subviral components such as empty virions, which may be produced late in an infection, some studies suggest that their practical level of sensitivity does not always exceed that of electron microscopy (*19*,*25*,*26*). Because this modern armament has taken over most routine diagnostics, with the exception of gastroenteric viral infections, electron microscopy may be concentrated on infectious disease emergencies. The “open view” of electron microscopic testing allows an unbiased, rapid detection of viruses and other agents if sufficiently high particle concentrations exist (Figure 1). Because of this capability, electron microscopic testing must be a frontline method, applied either to samples directly from a suspected lesion, bodily fluids, or biopsies after cell-culture augmentation of a cultivable agent or from letters and environmental samples.

## Specimen Collection

Successful investigation of any outbreak or novel case starts with specimen collection. Insufficient, improper, or inadequate sampling may delay or prevent identification of a causative agent. Sufficient sampling requires identification of, and sampling from, all areas where infection may have been established. Fecal samples are ideal for investigating gastroenteric episodes, as are lesion fluids or smears from skin lesions of possible viral origin.

**Figure 2:**
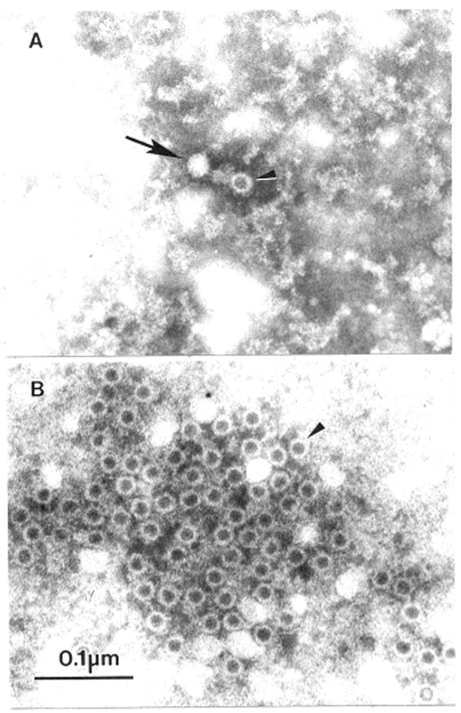
Association of human parvovirus B-19 with erythema infectiosum by immuno electron microscopic. A. Airfuge EM-90 rotor (Beckman, Palo Alto, CA) preparation of human serum prospectively collected at time of contact with case of erythema infectiosum. Erythema infectiosum-like rash developed 1 week after collection of serum. B. Immuno electron microscopic preparation of the serum in panel A. The serum was mixed with matched convalescent-phase serum (final dilution convalescent-phase serum 1:100), incubated for 90 min at 37°C, and virions/immune complexes centrifuged directly to a specimen grid with the EM-90 rotor. Arrow, complete virion, arrowhead, genome defective virion. phosphotungstic acid. Bar = 100 nm. For study details, see Plummer et al., 1985 (*16*).

A major cause of insufficient sampling can be failure to collect acute-phase sera from affected case-patients and their contacts. First, the existence of a blood-borne pathogen may not be evident when examining unexplained cases, as demonstrated by the difficulty identifying HIV (*27*) and hepatitis C virus infections, and associating human parvovirus B-19 with Fifth disease (*16*). Second, acute-phase sera are essential for demonstrating seroconversion to a suspected agent. Third, clinical symptoms may be caused by an immune response to an infection that has resolved by the time they appear. However, specimens from apparently uninfected contacts of patients with acute cases may contain the agent involved (*16*). Convalescent-phase sera collected from case-patients 4–6 weeks after onset of illness are also powerful diagnostic reagents. If no agent has been identified by standard virus detection procedures (e.g., electron microscopy, tissue culture, immunoassay, or nucleic acid amplification techniques), these serum samples may be used to detect the causative agent (*28*), while matched acute:convalescent-phase serum pairs collected at least 2 weeks apart may be used to demonstrate a significant rise in specific antibody among cases by immuno-electron microscopy (Figure 2) (*7*). Infectious agents may also be identified in cerebrospinal fluid, lesion crusts, nasopharyngeal washes, saliva, tears, urine, and biopsied tissue specimens (*29*). However, low viral load, sampling difficulties, or both may reduce the effectiveness of rapid electron microscopic diagnosis on these later types of specimens without initial tissue culture amplification, as observed in Nipah virus studies (*6*).

**Figure 3:**
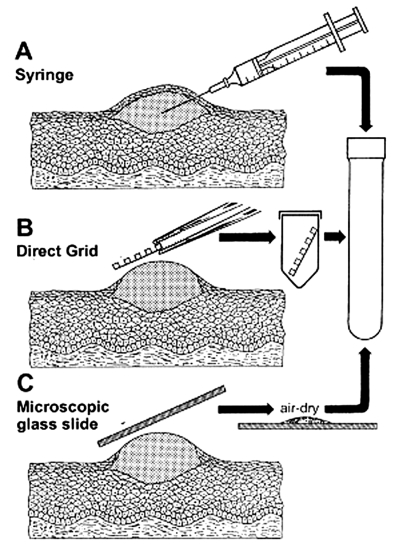
Three methods for efficient collection of vesicular and blister fluids for diagnostic electron microscopic. A. The contents of a vesicle are collected into the barrel of a needle. B. After the blister is opened, a coated electron microscopic grid is touched to the fluid and air-dried (direct electron microscopic). C. A glass microscope slide is touched directly to an unroofed lesion and a smear prepared. Samples are then placed in rigid containers for transport to the electron microscopic laboratory.

Safety concerns, miscommunication between infectious diseases specialists and staff who collect samples, or inadequate training may result in improper sample collection. Although swab samples placed into viral transport media may allow nucleic acid amplification techniques or culture of nonfastidious agents to be carried out; such specimens are not very conducive to successful rapid electron microscopic diagnosis of lesion exudates caused by dilution effects and interfering components. Several effective ways of collecting lesion fluids exist (*8*). A method readily available to the physician or in a hospital ward is collection into the barrel of a 26-gauge needle attached to a tuberculin syringe. A fresh lesion is unroofed or the beveled surface of the needle is placed against the base of an open lesion, and fluid is aspirated into the barrel. After capping, the sample may be transported directly for rapid electron microscope diagnosis (Figure 3A). Alternatively, coated electron microscopic specimen grids may be lightly touched directly to the vesicle fluid, lesion base, or both; allowed to air dry; and transported directly for examination (direct touch preparation) (Figure 3B). Because repreparing the sample with direct touch preparations may not be possible, at least two grids should be obtained when the specimen is collected. For safety and containment of hazardous infectious materials, the syringe or grid should be placed in a rigid sterile container, e.g., conical 15-mL centrifuge tube or Beem capsule (Beem Co., Bronx, NY), sealed with Parafilm (American National Can Co., Greenwich, CT), and the outside of the tube washed with 0.5% sodium hypochlorite (10% household bleach) before transport (Figure 3). Safety regulations usually require further packaging of the sample inside a second container.

In the late 1940s, direct touch preparations from skin lesions were prepared in North Africa and sent to Toronto, Canada, where they were examined successfully for smallpox virus for up to 4 months after collection (*11*). In another comparative study in Winnipeg, Canada, which used matched lesions, we observed an average increase of 10.2:1 in the number of virions visualized by direct touch as opposed to needle aspirate preparations, and the ratio was >1.0 in 92% of total cases examined (n=12; p<0.02; [Wilcoxon signed-rank test]). We observed no difference in the number of positive identifications or homogeneity of virion distribution on the grid between these two methods. Lesion smears on glass slides may also be used effectively for both electron microscopy and immunofluorescent microscopy examination (Figure 3C). Smears, i.e., dried down vesicle fluids, are especially effective when syringes and electron microscopic grids are not available. Both direct touch and smear preparations are useful when specimens must be transported some distance for electron microscopic examination.

Results from lesion exudates, collected as swab samples and placed in viral transport medium, are less useful. A change in specimen collection protocols in 1995, from direct touch/lesion aspirates to swab specimens in transport medium, has resulted in a decline in successful identification of virions in lesion specimens in Winnipeg from 62% to 75% to approximately 10% (Hazelton, unpub. data). While complete fecal samples are preferable, collecting rectal swab samples for diagnosis of gastroenteric agents may be necessary. These swab samples should be placed in capped conical centrifuge tubes with 0.2 mL sterile, distilled water, sealed with Parafilm, and sent for electron microscopic diagnosis. Lesion crusts should also be placed in sterile conical tubes. The addition of any liquid medium to lesion crusts, cerebrospinal fluid, nasopharyngeal washes, saliva, tears, and urine will not assist the electron microscope laboratory. Tissue biopsy samples in buffer without fixatives should be stored at 4°C and sent directly to both an electron microscope facility and a viral identification laboratory for rapid electron microscopy and other diagnostic testing. Fixation may interfere with antibody binding and thus preclude infectivity tests and successful application of any immunoelectron microscopy.

Finally, failure to collect an adequate volume of sample will limit the tests that may be used and the ability to successfully identify causative agents. Lesion fluids are deceiving. For example, samples containing poxvirus or varicella zoster virus that appear to have no material drawn into a needle barrel (Figure 3A) or attached to a grid may still contain numerous virions. When possible, at least 1 *g* of fecal material should be collected into a commercial stool collection vessel. A minimum of 5.0 mL of blood should be collected into tubes without anticoagulants. When a special interest in the case or outbreak occurs, large samples may provide reagents for later testing. All samples should be immediately sent for rapid electron microscopic diagnosis, with storage at 4°C if possible. Dried smears may be stored and transported at ambient temperature. Under no condition should samples be frozen for storage and transport before receipt at the diagnostic facility (*30*).

## Containment of Biological Hazards and Laboratory Safety

Protecting staff and containing infectious agents are important considerations in the handling of all clinical specimens. Samples may contain agents that are highly infectious or associated with a high mortality rate. In consideration of the possibility of bioterrorism and agri-terrorism, delivering samples to a central facility at biological safety level (BSL) 3 or higher may be necessary for inactivation before electron microscopic examination. Regardless, preparation must be done in a laminar flow hood with BSL-2 or greater containment capability. Most infectious agents may be inactivated in suspension by adding formaldehyde or glutaraldehyde (20 min, final concentration 2% and 0.5%, respectively). Alternatively, hazardous samples may be inactivated after they are mounted on the grid by treating the grid with fixative, by subjecting stained preparations to ultraviolet irradiation (UV) for 5 min before removing them from the biological safety cabinet, or both. UV treatment may, however, affect both virion morphology and grid stability. Prolonged treatment with glutaraldehyde or formaldehyde has little effect on morphology while inactivating most agents (*31*). Both formaldehyde and glutaraldehyde immobilize structures by Schiff reactions involving aldehyde side groups. As a di-aldehyde with a 5-carbon backbone, glutaraldehyde is more effective than formaldehyde at intermolecular crosslinking. Glutaraldehyde may, therefore, cause aggregation and obscure some fine structural detail. Samples suspected of containing spores should be inactivated with 10% formaldehyde final concentration because spores are more resistant to chemical inactivation (*32*). Specimens that may contain prions require more harsh treatment, such as the addition of 1 M NaOH, to inactivate the samples. However, treatment with NaOH will degrade most biologic structures to an indecipherable tangle of artefacts, and is, therefore, not conducive to electron microscopic examination.

Specimens that have not been inactivated must still be treated as potentially infectious after electron microscopic examination. For example, no decrease was observed in a 50% tissue culture infective dose (TCID_50_) of poliovirus samples after they were mounted on the grid and stained with 2.5 mM (1.6%) phosphotungstic acid, pH 7.0. Subsequent exposure to vacuum and the electron beam for 1 min reduced TCID_50_ by at least 10^6.5^ and 10^7.5^ for adenoviruses and polioviruses, respectively. More importantly, 10-min vacuum and electron beam exposure of grids containing sporulating *B. subtilis* preparations permitted colony recovery in 60% of tests and reduced colony-forming units 500-fold, and exposure to either vacuum or phosphotungstic acid-negative stain alone had little effect on the viability of adenovirus, poliovirus, or spore preparations (*33*). These observations underline the extreme resistance of spores in different weapons delivery systems. Because of the risk for residual infectivity, all grids must be disposed of as infectious waste, and equipment used to handle samples and grids, e.g., forceps, must be decontaminated by treatment with 5% glutaraldehyde for 20 min. Alternatively, equipment may be disinfected with 1 M NaOH. Cleaning is also necessary to prevent false-positive results caused by crossover contamination between specimens. Staff involved in rapid electron microscopy should be vaccinated for multiple agents, including smallpox and hepatitis B.

## Specimen Preparation

While rapid electron microscopy may be performed with any type of specimen, the requirement for truly rapid electron microscopic diagnosis is not common. Indicators include limiting exposure in clinically threatening situations in which an infectious cause is not ruled out, as may occur if a patient has suspected herpetic lesions in a ward for immunocompromised, newborn, or transplant patients; new clinical symptoms are observed with immunocompromised patients; the need to initiate early treatment; or the risk of passing infection during birth. Since a viral agent may be found by rapid electron microscopy in over 90% of poxvirus (*34*) and other skin lesions of viral etiology (Gelderblom and Hazelton, unpub. data), this method is ideal for investigating outbreaks of rash-like illness and suspected cases of bioterrorism.

**Figure 4:**
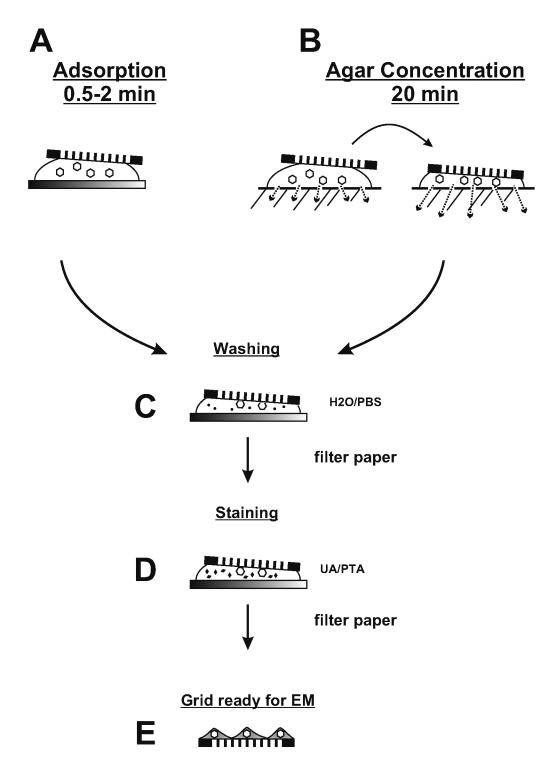
Two step staining preparation of suspension samples. Please see text for details.

A morphologic diagnosis may be obtained within 10 min of specimen arrival in the electron microscopic facility. The standard two-step drop method, i.e., adsorption followed by negative staining, is used for preparation (Figure 4A). Viral load is usually more than sufficient to allow successful diagnosis of herpesvirus, poxvirus, and some gastroenteric infections. Negative-stain examination is simple and may be conducted in any electron microscopic facility. The first item needed is a 400-mesh electron microscopic grid coated with either a single plastic layer or a plastic film reinforced with carbon (*32*,*35*,*36*). Carbon-coated plastic films have higher thermal stability and are less prone to specimen movement during examination. However, they may be more hydrophobic than plain plastic films. Electron microscope units that specialize in virus preparative or diagnostic techniques prepare their own plastic-coated, carbon-stabilized films, and glow discharge the films to improve hydrophilicity, particle adherence, and distribution of both sample and stain (*36*,*37*). Coated grids may also be purchased through most electron microscopy suppliers. Clinical samples with high concentrations of protein often do not require glow discharge pretreatment to reduce hydrophobicity.

Lesion fluids received in the barrel of a needle or capillary tube are expelled onto a hydrophobic surface such as Parafilm. If the sample has dried, a small drop of redistilled water (15 µL),

sterilized through a 0.2-µm–pore filter, is drawn into the specimen container and washed back out. If required, an aliquot of suspension should immediately be transferred to viral transport medium and submitted for cell culture, nucleic acid amplification techniques, and other virologic procedures. Lesion crusts and biopsy material may be soaked in 3 volumes buffer and solubilized by 10–12 pestle strokes in a Dounce homogenizer, while fecal material may be suspended by vortexing with glass beads in 3–9 volumes of distilled water. Heavy debris is allowed to settle, and the suspension cleared by low-speed centrifugation (1,000 x *g* for 5 min). Liquid samples (cerebrospinal fluid, nasopharyngeal washes, saliva, tears, and urine) may be used directly. If required, an equal volume of double concentration fixative may be mixed with the suspension to inactivate any infectious agents present before mounting the sample on the coated grid. A grid is floated with the coated surface on a drop of fixed suspension for 0.5–2 min and excess material wicked away with an edge of filter paper (Figure 4C). If bacteria are to be negatively stained, higher numbers of microorganisms will attach to the grid because of sedimentation when the drop is placed on the grid. Adsorption is not an absolute process. Any extra manipulations, such as washing the grid, may reduce the number of adsorbed particles. Pretreating the carbon-reinforced grids by glow discharge, poly-L-lysine, alcian blue, or UV light may also help for tighter binding (*32*,*35*) and are particularly useful when staining aldehyde-inactivated samples. Direct touch lesion fluid preparations, which are already mounted on the grid, may be rehydrated and inactivated before staining by floating the grid on a drop of fresh 2% formaldehyde.

Rapid immunologic methods that improve sensitivity when searching for unknown agents include solid-phase immunoelectron microscopic (SPIEM) (*38*) and serum in agar (SIA) (*39*), both of which may use either pooled human immunoglobulins (HuIgG) or specific antibodies. HuIgG may be obtained from most immunologic suppliers or hospital pharmacies. SPIEM concentrates antigens on the grid by immune capture, thereby improving the probability of observing an etiologic agent. The coated surface of a grid is floated on a drop of pooled HuIgG (100 µg/mL and 20 µg/mL in phosphoate-buffered saline [PBS] B) or antiserum (1/100 and 1/500 in PBS) for 10 min, washed on 6 sequential drops PBS, and floated on the specimen for 30–60 min at 37°C. The sample may be stabilized after SPIEM with 0.1% glutaraldehyde to ensure tight binding of the captured antigens, washed on 6 drops of PBS, negative stained, and examined (*38*). SIA uses immunoaggregation to identify antigens. In addition, type-specific antisera may be used in SIA to serotype the agent present. Antibody (1/100 for antisera and 100 µg/mL for HuIgG) is prepared in cooled 1% agar. A grid is placed on the solidified agar, and a drop of sample placed over the grid. Diluent diffuses into the agar while antibody diffuses into the suspension and antigen:antibody complexes form, which then adsorb to the grid as diluent volume is reduced (Figure 4B) (*39*).

## Negative Staining

**Figure 5:**
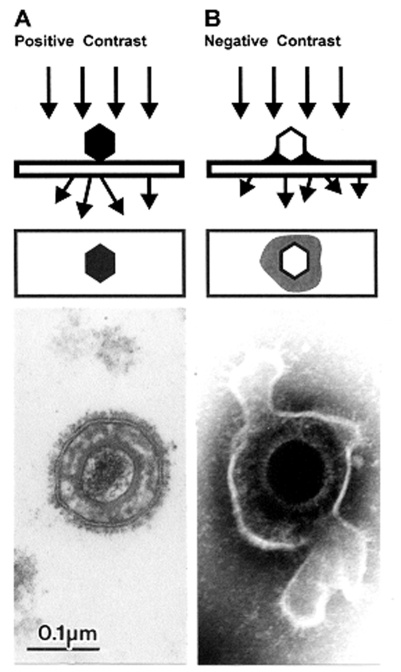
Comparison of herpesvirus appearance after positive and negative stain electron microscopic. A. Positive staining. Samples undergo a lengthy process of fixation, incubation with heavy metal ions (osmium, uranyl), dehydration, embedment, ultrathin sectioning, and staining. Chemical moieties in the object show differential affinities for the heavy metal stains, resulting in a clear outline of the viral bilayer envelope, viral envelope proteins, nucleocapsid, and the dense nucleic acid containing core. B. Negative staining. After a brief fixation, samples are mounted directly on electron microscopic grids and stained as in Figure 4. The electron-dense stain (phosphotungstic acid [phosphotungstic acid], uranyl acetate, and the like) penetrates the virion and embeds the particle in a matrix of stain. Due to density differences between the stain and weakly scattering biological components of the virion, the virion appears as a transparent and detailed reverse (negative) image. Penetration of stain into the nucleocapsid provides a dense core with the crenellated appearance presented by the central channel of capsomers on the nucleocapsid surface. Viral surface proteins appear as projections from the labile envelope. phosphotungstic acid. Bar = 100 nm.

Biologic structures, because of low mass density, interact weakly with electrons used for imaging, and therefore, show little contrast or detail. Several ways exist to generate sufficient image contrast and resolution; the most versatile is positive and negative staining with heavy metal ions, e.g., lead, tungsten, and uranium ions. Positive staining depends on chemical reactivity with the components of the object and involves fixation, postfixation, embedding in resins, ultrathin sectioning, and multiple staining incubations. These procedures may take 4–5 days before a sample is ready for examination. Rapid embedding protocols can reduce the time to approximately 1 day but with a loss in specimen quality (*32*). In contrast, negative staining is simple, rapid, and well suited for examination of small particulate suspensions. A coated grid with sample adsorbed to the surface is floated on a drop of negative stain for 0.5–2 min, excess stain wicked away with a piece of filter paper, air dried for 1–3 min, and examined by electron microscopy (Figure 4D). Structures on the grid are surrounded and stabilized by the drying stain. Thus, they appear as transparent, highly detailed negative images within a dark halo of stain (Figure 5B).

The most common negative stains are 1% (60 mM) aqueous uranyl acetate, pH 2-4.5, and 1% (2.5 mM) phosphotungstic acid, pH adjusted to 7.0 with NaOH. Aqueous uranyl acetate is unstable at higher pH values. Because aqueous uranyl acetate and phosphotungstic acid differ in staining properties, both stains should be applied in parallel in case of unknown samples. Stains should be relatively fresh and stored in brown glass bottles at 4°C (*32*,*35*,*36*). While examining the stained grid, additional grids may be left floating on the sample droplet, protected from dust and drying. This method reduces preparation time in the event additional grids must be prepared for electron microscopic inspection.

## Particle Enrichment

If no virus has been identified after 20 min or after the examination of 10 grid squares, the result may be considered to be no etiologic agent identified. Routine two-step drop preparations for electron microscopic diagnostic procedures require particle concentrations of 10^6^ to 10^8^/mL. Therefore, negative evidence is not an absolute diagnosis. A number of effective concentration or immunologic procedures exist that markedly increase sensitivity of electron microscopic diagnostics for samples with lower particle concentrations (*32*,*40*). These procedures take from 0.5 to 16 hours and are labor and training intensive. Viral research or diagnostic facilities generally have access to at least one advanced procedure. Nonimmunologic procedures include: a) ultracentrifuge concentration—the material from cleared suspensions is sedimented by ultracentrifugation, resuspended in a smaller volume and then prepared by the standard two-step drop method (*32*); b) agar diffusion—a 20–50 [20- to 50-µL drop of suspension is placed on 1% agar. As the fluid is absorbed the virus is concentrated. After 15–20 min, a grid is placed on the remaining suspension and then stained as with the two-step method above (Figure 4D). This procedure will result in an enrichment factor of approximately 5x (*32*); and c) direct centrifugation to the electron microscopic grid with the Beckman Airfuge (Beckman, Palo Alto, CA) EM-90 rotor or A-100 rotor, a procedure that increases sensitivity up to 1,000 fold (*40*–*42*). Immunoaggregation and immunodecoration with type- and genus-specific antibody may be used to concentrate material or to specifically identify the agent, e.g., herpes simplex 1 and 2 and varicella zoster. Also, convalescent-phase serum samples may be used to identify infectious agents or provide evidence of seroconversion to the agent when paired with acute-phase sera. For standard immunoelectron microscopy, the suspension is incubated for 1 h at 37°C with serum samples diluted in PBS, and then mounted on the grid by using either the drop method or direct centrifugation to the grid. Immunoaggregation may be very powerful in the identification of a suspected or novel agent or with small, dispersed virions (*7*,*13*,*16*). Immuno electron microscopy was particularly useful in the initial identification of noncultivable agents such as hepatitis C, Norwalk virus, and Winnipeg virus (*7*,*13*,*43*). Detailed methods may be found in references (*29*,*32*,*35*,*44*).

As with all diagnostic laboratory procedures, diagnostic electron microscopy should be performed in a quality-controlled manner. For routine external quality control, the Konsiliarlaboratorium für EM-Erregerdiagnostik at the Robert Koch-Institut in Berlin has conducted an External Quality Assurance-EM Virus Program, which provides panels of specimens containing different agents, since 1994. More than 95 laboratories from 27 countries participated in EQA-EMV 11 during August and September 2001. Each laboratory used its preferred method for preparation (*45*). A review of results submitted from participating facilities indicated that 27 of 69 laboratories correctly identified all test samples, while an additional 28 successfully identified four of five positive specimens. A trend towards higher success existed among laboratories that used enrichment procedures (35 of 55) when compared with those that were less successful (4 of 14) (p=0.055). However, experience, as defined by years of service and number of samples examined annually, was another important success factor.

## Identification of Viral Agents

**Figure 6:**
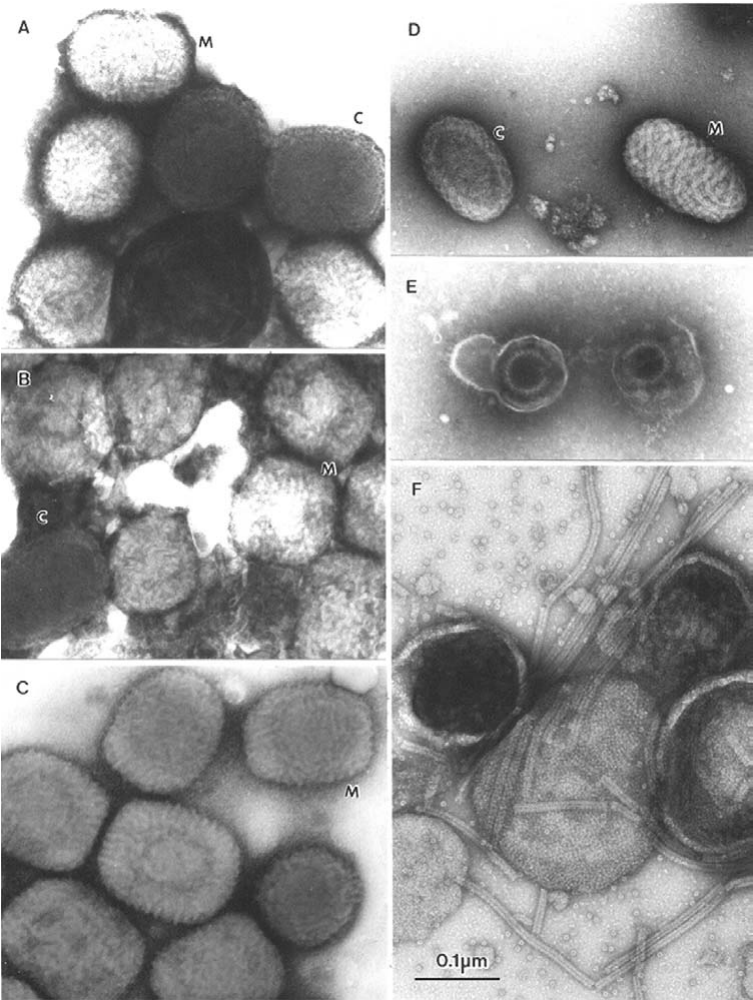
A–E. Comparison of clinically relevant viral agents associated with skin lesions. A–C show poxviruses indistinguishable in appearance from variola virus, the agent of smallpox. The slightly rounded, brick-shaped virions measure about 270 by 350 nm. Two types of particles may be seen. M, or Mulberry forms show a 10- to 20-nm diameter short-tubular or beaded surface (M). Capsular, or C forms, partly penetrated by the stain, are recognized by a 30-nm membrane (C): A. Molluscum contagiosum (molluscipoxvirus) virions from skin lesions observed in an adult; B. Vaccinia virus vaccine strain WR (orthopoxvirus) from cell culture; C. Ectromelia virus (orthopoxvirus) from culture material. D. Parapox viruses measure up to190 by 300 nm and are more distinctly ovoid. Tubules, 10 to 20 nm wide and approximately 1,000-nm long, spiral around the virion, giving a distinctive crosshatched appearance. E. Herpesvirus particles from a skin lesion of a primary varicella zoster infection observed in an adult. Direct electron microscopic shows two virions. The envelopes are broken, liberating the 100-nm nucleocapsid. F. Cell culture supernatant from a patient with an infantile respiratory tract infection. The enveloped virions are studded with tiny surface spikes. The 18-nm helical nucleocapsids have been released from disintegrating virions. The nucleocapsids and envelope details are typical of paramyxoviruses. A–B, phosphotungstic acid, C–F, uranyl acetate. All prints at the same magnification, bar = 100 nm.

Several major pitfalls exist in the identification of viral agents by negative stain electron microscopy. First, the failure to detect and identify an agent does not mean that it is not there. Second, if you look long enough and hard enough, you will eventually find something that resembles what you wish to find. Third, the presence of a single picture cannot validate the interpretation of morphology. While the diagnostician must not be afraid to find something novel, the finding must be real. One example is the observation of multiple particles with similar morphology. In addition, photographic records must be made for all possible positive identifications and reviewed to confirm the accuracy of the initial diagnosis. Further, when a particle is assigned to a proper virus family, reviewing the case may be necessary to identify the genus or strain. For example, not all samples with orthopoxvirus morphology will be smallpox (Figure 6). While natural infections of variola virus have been eradicated, many other orthopoxvirus continue to be found and identified, e.g., camel-, cow-, monkey-, mouse-, and vaccinia pox viruses (*17*,*46*). In addition, the molluscipoxvirus Molluscum contagiosum is morphologically indistinguishable from orthopoxviruses. Identification of Molluscum contagiosum was essentially non-existent in Winnipeg before 1983. With the growth of the immunocompromised sector of the population, the number of identifications increased to 6–10 cases per year until 1995, when the change in sampling methods from lesion aspirates to swab collection in transport medium resulted in a reduction to 1–2 Molluscum contagiosum identifications per year (Hazelton, unpub. data). Further differentiation of poxviruses into variola, vaccinia, cowpoxviruses, or Molluscum contagiosum may be performed by immuno electron microscopy with type-specific antibodies. Appropriate antibodies and the latest nucleic acid amplification techniques are also available for this determination at the World Health Organization Collaborating Centers at the Centers for Disease Control and Prevention, and VECTOR, Koltsovo, Novosibirsk Region, Russia.

## Future Impact of Diagnostic Electron Microscopy

Compared with other laboratory diagnostic methods, electron microscopic excels with respect to rapidity and the open view that permits detection and identification of both novel agents and those not considered by the clinician. However, full exploitation of this potential requires early and coordinated application of electron microscopy with other frontline diagnostic procedures. The use of electron microscopy to examine diagnostic cultures of Hendra virus provided evidence of a paramyxovirus 3 days before any other results were available. Thus, focusing further characterization on the proper virus family was possible, and a novel pathogenic agent, which became the prototype strain for the henipah viruses, a proposed new genus of paramyxoviruses, was found (*17*,*47*). Diagnostic electron microscopy does not need to be either expensive or difficult to perform if executed in a diagnostic network, i.e., by recruiting instruments and electron microscopists working in other departments, e.g., cell biology or pathology (*48*). Respective arrangements are facilitated by using inactivated samples and implementing new technologies, such as automated pattern recognition (*49*) and telemicroscopy by using digital image acquisition and remote operation of the instrument or review of micrographs through the Internet (*50*).

**Figure 7:**
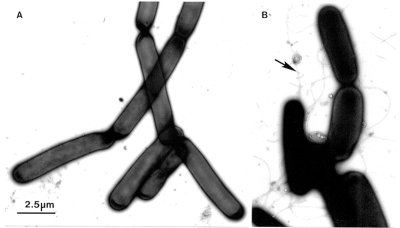
A. A colony of *Bacillus anthracis* was suspended, inactivated, and negatively contrasted with aqueous uranyl acetate, as described for Figure 4. The microorganisms, which grow in long chains, do not have flagella. B. The ubiquitous *B. subtilis* may also grow as long chains. However, in contrast to *B. anthracis*, the *B. subtilis* cells show distinct flagella (arrow). Bar = 2.5 μm.

As smallpox diagnosis from the 1940s to the 1970s, electron microscopy differential diagnosis has often ruled out the occurrence of dangerous pathogens. The power to rapidly identify agents of bioterrorism has now been demonstrated convincingly by Tom Geisbert and Peter Jahrling, U.S. Army Medical Research Institute of Infectious Disease, when they identified and quantified spores in the *B. anthracis* bioterrorist letter attack upon U.S. Senate Majority Leader Daschle (Jahrling, pers. commun.) (Figure 7). Because the unusual and unexpected can be rapidly identified, electron microscopy must remain a frontline method for rapid diagnostic virology, investigation of potential bioterrorist events, and investigation of new and unusual cases of suspected infectious origin.

## Supplementary Material

Appendix Directory of electron microscope facilities which may provide assistance and advice concerning emergency viral diagnostic matters.^1^
