# Supplementary material for: Electron Microscopy for Rapid Diagnosis of Emerging Infectious Agents
Source: Emerg Infect Dis. 2003 Mar;9(3):294–303. doi: 10.3201/eid0903.020327 (PMC2958539; doi:10.3201/eid0903.020327)
Supplement: Appendix — Directory of electron microscope facilities which may provide assistance and advice concerning emergency viral diagnostic matters.1 [file 02-0327_App-s1.pdf]

Appendix. Directory of electron microscope facilities which may provide assistance and advice concerning emergency viral diagnostic matters.[1]

Australia
Alex Hyatt
Geelong, Victoria, Australia
Phone: 61 0352275419
Fax: 61 0352275555
e-mail: alex.hyatt@csiro.au

John Marshall
North Melbourne, Victoria, Australia
Phone: 61 3 93422678
Fax: 61 3 93422660
e-mail: john.marshall@mh.org.au

Austria
Wolfgang Muss
Salzburg, Austria
Phone: 43 662-4482-4720
Fax: 43 662-4482-882
e-mail: W.Muss@lks.at

Susanne Richter
Wien, Austria
Phone: 43 173216/5146, 47 173216/5107
Fax: 43 173216/5194
e-mail: Susanne.richter@bfl.gv.at

Belgium
Patrick Goubau
Brussels, Belgium
Phone: 32 02 7645492
Fax: 32 02 7645422
e-mail: goubau@mblg.ucl.ac.be; laboratoire.sida@mblg.ucl.ac.be

Marc van Ranst
Leuven, Belgium
Phone: 32 16 332160
Fax: 32 16 337340
e-mail: Marc.Vanranst@rega.kuleuven.ac.be

[1] Do not send samples to any facility without first contacting the facility for instructions; not all labs listed meet biological safety level 3 conditions; facilities may be required to refer queries to other, jurisdictional laboratories.

Brazil

Hermann Schatzmayr
Rio de Janeiro, Brazil
Phone: 55 21 598 4274
Fax: 55 21 270 6397
e-mail: Hermann@ioc.fiocruz.br

Marli Ueda
São Paulo, Brazil
Phone: 55 11-3068-2908
Fax: 55 11-3088-3753
e-mail: marliueda@hotmail.com

Canada

Hans-Wolfgang Ackermann
Laval, PQ, Canada
Phone: 1 418-656-2131, ext. 2558
Fax: 1 418-656-7666
e-mail: Ackermann@mcb.ulaval.ca

Paul Hazelton
Winnipeg, MB, Canada
Phone: 1 204-789-3313
Fax: 1 204-789-3926
e-mail: paul_hazelton@umanitoba.ca

Judith Isaac-Renton
Martin Petric
Vancouver, B.C. Canada
Phone: 1 604-660-6032
Fax: 1 604-660-6073
e-mail: judy.Isaac-renton@bccdc.ca

Raymond Tellier
Toronto, Ontario, Canada
Phone: 1 416-813-6592
Fax: 1 416-813-6257
e-mail: Raymond.tellier@sickkids.on.ca

Don Stoltz
Halifax, NS, Canada
Phone: 1 902-494-2590
Fax: 1 902-494-5125
e-mail: dstoltz@is.dal.ca

Czech Republic
Jana Schramlová
Prague, Czech Republic
Phone: 420 2 6708 2572
Fax: 420 2 7274 4354; 420 2 6708 2387
e-mail: jschraml@szu.cz

Denmark
Jens Blom
Copenhagen, Denmark
Phone: 45 3268 3578
Fax: 45 3268 3883
e-mail: jbl@ssi.dk

Finland
Olli Vapalahti
Helsinki, Finland
Phone: 358 9-19126604
Fax: 358 9-19126491
e-mail: olli.vapalahti@helsinki.fi

Carl-Henrik von Bonsdorff
Helsinki, Finland
Phone: 358 9-1912-6506
Fax: 358 9-1912-6491
e-mail: carl-henrik.vonbonsdorff@helsinki.fi

France
Pierre Gounon
Nice, France
Phone: 33 04 92 07 60 46
Fax: 33 04 92 07 60 45
e-mail: gounon@unice.fr

Germany
Stefan Becker, Larissa Kolesnikova, Hans Dieter Klenk
Marburg, Germany
Phone: 06421-2865433
Fax: 06421-2865482 or 06421-2868962
email: Stefan Becker becker@mailer.uni-marburg.de
Larissa Kolesnikova kolesnick@mailer.uni-marburg.de
Hans Dieter Klenk klenk@mailer.uni-marburg-de

Werner Eichhorn, Oskar-R. Kaaden
München, Germany
Phone: Eichhorn 49 89-2180 2531

Kaaden 49 89-2180 2535
Fax: 49 89-2180 2597
e-mail: werner.eichhorn@micro.vetmed.uni-muenchen.de

Bernhard Fleischer, Herbert Schmitz, Christel Schmetz
Hamburg, Germany
Phone: 49 40 42818 467, 49 40 42818 468
Fax: 49 40 42818400
e-mail: pcs@bni.uni-hamburg.de; bni@bni-hamburg.de

Hans Gelderblom, Reinhard Kurth, Georg Pauli, Andrea Männel
Berlin, Germany
Phone: H. Gelderblom 49 30-4547-2337
A. Männel 49 30-4547-2326
Fax: 49 30-4547-2334
e-mail: gelderblomh@rki.de

Harald Granzow, Th. C. Mettenleiter
Insel Riems, Germany
Phone: 49 38 351-7206
Fax: 49 38 351-7151
e-mail: Harald.Granzow@Rie.BFAV.de

Bärbel Hauröder
Koblenz, Germany
Phone: 49 261 896-7260
Fax: 49 261 896-7109
e-mail: b.hauroeder@zinstkob.de

K.-F. Reckling
Stendal, Germany
Phone: 49 3931-631818
Fax: 49 3931- 631 153
e-mail: Reckling@lvluasdl.ml.lsa-net.de

India

Atanu Basu
Pune, India
Phone: 91 20-6127301
Fax: 91 20-6122669
e-mail: Atanu Basu atanu_b@hotmail.com
Milind Gore milind_gore@hotmail.com

Ireland

WW Hall, Patrick Costigan
Dublin, Ireland

Phone: 353 1-716-1338, 353 1 716-1354
Fax: 353 1-269-7611
e-mail: patrick.costigan@ucd.ie

Israel
Jossi Manor
Hashomer, Israel
e-mail: ymanor@sheba.health.gov.il

Italy
Guisy Cardeti, Nazareno Brizioli
Roma, Italy
Phone: 39 06 79099448
Fax: 39 06 97340724
e-mail: gcardeti@rm.izs.it

Carlo Chezzi
Parma, Italy
Phone: 39 0521-988885
Fax: 39 0521-993620
e-mail: clchezzi@ipruniv.cce.unipr.it

Massimo Gentile
Roma, Italy
Phone: 39 06 447441224
Fax: 39 06 447441236
e-mail: gentilemax@tiscalinet.it

Antonio Lavazza, Ezio Lodetti
Brescia, Italy
Phone: 39 30 229 0298
Fax: 39 30 242 5251
e-mail: alavazza@bs.izs.it

Japan
Toshiyuki Goto
Kyoto, Japan
Phone: 81 75 751-3925
Fax: 81 75 751-3909
e-mail: tgoto@itan.kyoto-u.ac.jp

Naomi Sakon
Osaka, Japan
Phone: 81 6 6972-1321
Fax: 81 6 6972-2393
e-mail: sakon@iph.pref.osaka.jp

Etsuko T. Utagawa
Tokyo, Japan
Phone: 81 3 5285-1111
Fax: 81 3 5285-1161
e-mail: etu@nih.go.jp

Netherlands

Albert DME Osterhaus
Rotterdam, Netherlands
Phone: 31 10-4088066
e-mail: Osterhaus@viro.fgg.eur.nl

Paul Roholl
Bilthoven, Netherlands
Phone: 31 30 2743651
Fax: 31 30 2744437
e-mail: p.roholl@rivm.nl

Russia

Elena Ryabchikova
Koltsovo, Novosibirsk Region, Russia
Phone: 383 2-36-60-01
Fax: 383 2-36-74-09
e-mail: lenryab@vector.nsc.ru

Slovenia

Mateja Poljsak
Ljubljana, Slovenia
Phone: 386 1 5437460
Fax: 386 1 5437401
e-mail: mateja.poljsak-prijatelj@mf.uni-lj.si

Spain

Maria Inmaculada Herrera
Majadohonda/Madrid, Spain
Phone: 91 509 7969/01
Fax: 91 509-7966
e-mail: iherrera@isciii.es; alvarezh@teleline.es

Sri Lanka

Mohamed Abdul Azeez Razak
Colombo, Sri Lanka
Phone: 94 1-693532, 1-693533, 1-693534, ext. 411
Fax: 94 1-575405
e-mail: azeezrazak@hotmail.com, medresit@slt.lk

Switzerland

Monika Engels, P. Wild, Elisabeth M. Schraner
Zurich, Switzerland
Phone: 41 1 635 8791
Fax: 41 1 635 8911
e-mail: emschra@vetanat.unizh.ch

Thomas Baechi
Zürich, Switzerland
Phone: 41 1 634 26 65
Fax: 41 1 634 49 06
e-mail: baechi@emz.unizh.ch

United Kingdom

A. Barry Dowsett
Porton Down, UK
Phone: 44 0 1980 612247
Fax: 44 0 1980 611096
e-mail: barry.dowsett@camr.org.uk

Bill Cooley
Addlestone, United Kingdom
Phone: 44 0 1932 357824
Fax: 44 0 1932 357659
e-mail: w.a.cooley@vla.defra.gsi.gov.uk

United States

John Bozzola
Carbondale, IL, USA
Phone: 1 618-453-3730
Fax: 1 618-453-2665
e-mail: bozzola@siu.edu

Joan Dragavon
Seattle, WA, USA
Phone: 1 206-341-5210
Fax: 1 206-341-5237
e-mail: dragavon@u.washington.edu

Carol E. Hearne, Donal O'Toole
Laramie, WY, USA
Phone: 307-742-6638
Fax: 307-721-2051
e-mail: CEHearne@uwyo.edu
Donal O'Toole@wyo.edu

Charles Humphrey, Cynthia S. Goldsmith
Atlanta, GA, USA
Phone: Charles Humphrey 1 404-639-3307
Cynthia Goldsmith 1 404-639-3306
Fax: 1 404-639-3043, 404-639-1377
e-mail: Charles Humphrey: cdh1@cdc.gov
Cynthia Goldsmith: csg1@cdc.gov
Peter Jahrling
Frederick, MD, USA
Phone: 1 301-619-2772
Fax: 1 301-619-4625
e-mail: peter.jahrling@det.amedd.army.mil

Sara E. Miller
Durham, NC, USA
Phone: 1 919-684-3452
Fax: 1 919-684-3265
e-mail: saram@duke.edu

Frederick A. Murphy
Davis, CA, USA
Phone: 1 530-754-6175
Fax: 1 530-752-2801
e-mail: famurphy@ucdavis.edu

Robert W. Nordhausen
Davis, CA, USA
Phone: 1 530-752-8760
Fax: 1 530-752-6253
e-mail: rwnordhausen@ucdavis.edu
